# Health-Related Quality of Life Among Ukrainian War Refugees Compared to the General Population in Estonia

**DOI:** 10.3389/ijph.2026.1608807

**Published:** 2026-02-05

**Authors:** Rainer Reile, Johann Saavaste, Galina Opikova, Taavi Lai, Juanita Haagsma

**Affiliations:** 1 National Institute for Health Development, Tallinn, Estonia; 2 Fourth View Consulting, Tallinn, Estonia; 3 Erasmus University Medical Center, Rotterdam, Netherlands

**Keywords:** EQ-5D, Estonia, health-related quality of life, refugee, Ukraine

## Abstract

**Objectives:**

The study aimed to provide a comparative analysis of HRQoL and its health-related and socio-demographic correlates among Ukrainian refugees and general population in Estonia.

**Methods:**

Study used age and sex matched data (1249 pairs) from two representative cross-sectional surveys covering Ukrainian refugees aged 18–64 years residing in Estonia (n = 1,430), and the general population (n = 2007) of Estonia in 2024. Tobit-regression was used to compare the EQ-5D-3L index values in both groups while controlling for wide range of socio-demographic and health indicators.

**Results:**

Refugees reported less restrictions in mobility, self-care or in performing usual activities, whereas higher prevalence of pain/discomfort and anxiety/depression was found for refugees compared to control group. Refugees had slightly lower EQ-5D index score (estimate −0.017, p = 0.029) compared to population controls after adjustment for socio-demographic and health-related covariates.

**Conclusion:**

Variations in EQ-5D-3L dimensions and index scores between refugees and population controls contribute to the literature on refugee HRQoL and extend the knowledge on HRQoL of Ukrainian refugees in the context of ongoing refugee crisis in Europe while also improving knowledge for support provision to this refugee group in Estonia.

## Introduction

According to the United Nations [1], over 5.3 million inhabitants have left Ukraine for other European countries following the Russian aggression in February 2022. These displaced people have endured high psychosocial stress, which coupled with migratory status, and lack of social and economic safety nets in the new setting are likely to have direct health impacts [2] even though host countries often provide extended health and social care coverage to the refugees. Available evidence suggests that while the health needs of refugees are diverse and complex, mental healthcare, preventive services and long-term care are among top priorities [3]. However, there is persisting need for better data on refugees’ health status and health needs to tailor support to the needs of the specific refugee groups.

Health-related quality of life (HRQoL) is an important measure of health as it encompasses not only self-reported physical health but also mental, emotional, and social dimensions of wellbeing. HRQoL among refugees is generally lower than that of the general population of the host countries [4]. The reduced HRQoL has often been associated with high exposure to potentially traumatic experiences before or during the displacement [5, 6] but difference in HRQoL can also be explained by poorer physical and mental health [4]. Refugees may face difficulties in accessing the healthcare system which potentially translates to strong link between HRQoL and social integration reported in earlier studies [4, 6]. As demonstrated in previous studies [7, 8], the socio-demographic differences between refugees and host population–related mostly to the sex- and age distribution but also to the socioeconomic status–contribute additionally to HRQoL differences. This disparity in HRQoL has significant implications for the health and social care systems of host countries, potentially increasing the demand for medical and psychological support services [9].

The escalation of the Russo-Ukrainian war in 2022 caused a large-scale flow of refugees both originating and resettling within Europe. This contrasts with the experience from the refugee crisis following the Syrian war little more than a decade ago [10], when larger differences in socio-cultural context might have had varied public health implications compared to current situation. Although a recent experimental study [11] has demonstrated consistent and relatively high support for migrants irrespective of background across Europe, several others [12, 13] have associated refugees originating from Ukraine with more supportive public attitudes. Given that a systematic review by Gagliardi et al [6] has associated lower HRQoL scores with difficulties in accessing and understanding the new healthcare system, community loss and cultural gap experienced in the new country, it is plausible that available evidence on differences of refugee and host populations’ HRQoL might not fully apply in the context of Ukrainian refugees. So far only a few studies [14–16] have examined HRQoL in this population and indicate lower HRQoL among Ukrainian refugees, which varies by sociodemographic and mental health indicators. However, the aforementioned studies rely on convenience sampling, which may limit the generalizability of the findings. To the best of our knowledge, there have been no studies that allow direct comparisons of the HRQoL of Ukrainian refugees and that of the general population of the host country using a matched case-control design to reduce the confounding due to socio-demographic differences between the refugees and the general population.

This study will focus on Estonia, where by early 2024 approximately 32,500 refugees from Ukraine had been formally registered, constituting about 2.4% of Estonia’s population (1.37 million) in January 2024 [17]. Given the extension of national healthcare coverage to registered refugees, health and social services should be available to all in case of need. According to United nations report, 15%–20% of Ukraine refugees residing in Estonia had specific needs and every third had experienced health problems in 2024. While the report [18] also confirmed that healthcare was received when needed in almost nine out of ten cases, an in-depth analysis of HRQoL profile of refugee population can significantly contribute to further policy and service delivery planning to address the perceived needs of the refugees in Estonia. To fill the evidence gap considering HRQoL of Ukrainian refugees, the study aims to compare HRQoL of refugees to the general population in Estonia.

## Methods

The study combined data from two population health surveys conducted in 2024 covering: a) Ukrainian refugees residing in Estonia, and b) Estonian general population. The survey on Health and wellbeing of Ukrainian refugees in Estonia (SHURE) was based on a random sample of 4000 individuals aged 18–64 who had been granted refugee status since 24th February 2022 according to the National Population Registry. Data on general population originates from the 18th wave of Health Behaviour among Estonian Adult Population (HBEP) [19]. This biennially repeated cross-sectional survey was based on a representative sample of 16–64-year-old Estonian residents (n = 5,000). Both surveys used mixed-mode (online and postal questionnaires) method and were carried out between March to June 2024. The questionnaires were harmonized, providing comparable data on HRQoL and its correlates for both study populations.

SHURE data included 1,430 responses (334 males and 1,096 females, crude response rate 35.8%) whereas HBEP survey resulted in 2007 responses (835 males and 1,172 females, crude response rate 40.1%). Given the statistically significant differences in demographic distributions between two populations, case-control matching procedure (1:1 exact matching based on sex and age) was applied. Resulting sex and age-matched dataset comprised of 1,249 refugees and 1,249 population controls aged 18–64 years and forms the analytic sample for this study.

EuroQol’s EQ-5D-3L was used to assess HRQoL in both study populations. This widely used generic health status measure includes five dimensions (mobility, self-care, usual activities, pain/discomfort and anxiety/depression) evaluated on a three-level scale [20]. The resulting health state description can provide 243 unique health profiles that can be transformed into country-specific index scores. As EQ-5D-3L based value sets for Estonia are not available, European value set [21] constructed using VAS valuation data from 11 valuation studies in 6 European countries, was used to derive HRQoL index values.

Both EQ-5D descriptive system and index values were used to compare the refugees and their population controls. Additionally, several socio-demographic and health-related indicators were included in the analysis. For sex, dichotomous classification (male, female) was used. Age (in full years) was used as a continuous variable in modelling whereas descriptive analysis used categorical variable (18–24, 25–34, 35–44, 45–54, 55–64-year). Current marital status was categorized as single, married/cohabiting, divorced/separated. Educational level refers to the highest level of education obtained and was aggregated into three groups: primary or lower, secondary/vocational, and tertiary/higher education. Income was based on average monthly net income per household member and categorized as: <900, 900–1,299, 1,300–1,700, and >1,700 Euros. Additional indicator on household’s financial wellbeing during the past month included categories: living comfortably, coping, finding it difficult, finding it very difficult. For health-related variables, self-rated health (good, average, poor) and three indicators on mental health were included. Perceived stress was assessed with a question: “In the past 30 days, have you been stressed, under pressure?” with response options dichotomized as yes/no. Depressiveness was addressed with a question: “In the past 30 days, have you been unhappy, depressed (suffering from depressiveness)?” with dichotomized categories yes/no being used in the analysis. Overtiredness was assessed with a question: “In the past 12 months, how often have you felt overtired?” with response options “almost all the time” or “quite often” referring to being overtired and “seldom” or “never” for no overtiredness.

Descriptive statistics including proportions and their 95% confidence intervals (95% CIs) were used to compare HRQoL by socio-demographic and health indicators in both study populations. Group differences in EQ-5D-3L domains and index values were assessed using z-test with Bonferroni correction applied to account for multiple testing [22]. Given the EQ-5D-3L index values are positively skewed and have a ceiling effect, we used Tobit regression. This regression technique is suitable for such data [23] and was used to compare the EQ-5D-3L index between Ukrainian refugees and population controls. First, univariate models were run for all independent variables. This was followed by Model 2 where study group variable was additionally adjusted to sex, age, marital status and education. Model 3 included variables from Model 2 and introduced two variables reflecting economic situation. In Model 4, health-related indicators were additionally included. Finally, statistically non-significant variables were sequentially excluded from Model 4 starting from the highest p-value until there were none left which resulted in Model 5. No substitution of missing values was used and the subset with 2180 observations with complete data for all included variables was used in regression models. The results were presented as beta coefficients, indicating the mean change in the reference value within the variable along with the p-values. The analyses were performed using statistical software R 4.3.1 and SPSS 29.0 (IBM Corp).

## Results

Table 1 presents the socio-demographic and health-related characteristics of the analytical sample. After matching, the sex and age distribution of Ukrainian refugees and general population were identical, but several differences in sociodemographic and health indicators remained statistically significant. Most notably, higher proportion of divorced/widowed and those with tertiary education were found for refugee study group compared to population controls. Substantial differences were also present for both income indicators: 22.9% of controls reported a monthly income exceeding 1700 euros, compared to only 3.6% among refugees and current household subsistence level was assessed as difficult (25.6%) or very difficult (9.4%) by significantly more individuals compared to control group (20.1% and 5.2%, respectively). Statistically significant differences were also found by health indicators with good self-rated health reported by 51.1% of refugees and 59.4% of controls whereas reported stress was more prevalent among the general population (28.5%) compared to refugees (21.7%).

**TABLE 1:** Characteristics of the study sample by socio-demographic and health variables (Estonia, 2024).

Variable	Category	Age & sex matched case-control data			
		Refugee cases		Population controls	
		n	% (95% CI)	n	% (95% CI)
Total	​	1,249	100	1,249	100
Sex	Male	334	26.7 (24.3–29.2)	334	26.7 (24.3–29.2)
	Female	915	73.3 (70.8–75.7)	915	73.3 (70.8–75.7)
Age	18–24	176	14.1 (12.2–16.1)	176	14.1 (12.2–16.1)
	25–34	290	23.2 (20.9–25.6)	290	23.2 (20.9–25.6)
	35–44	365	29.2 (26.8–31.8)	365	29.2 (26.8–31.8)
	45–54	274	21.9 (19.7–24.3)	274	21.9 (19.7–24.3)
	55–64	144	11.5 (9.8–13.4)	144	11.5 (9.8–13.4)
Marital status	Single	251	22.7 (20.3–25.2)	301	24.2 (21.9–26.6)
	Married/cohabiting	695	62.7 (59.8–65.5)^a^	829	66.6 (64.0–69.2)^a^
	Divorced/widowed	162	14.6 (12.6–16.8)^a^	114	9.2 (7.7–10.9)^a^
Education	Primary or less	46	3.7 (2.8–4.9)^a^	43	3.5 (2.5–4.6)
	Secondary/vocational	617	49.8 (47.0–52.5)^a^	730	58.6 (55.8–61.3)^a^
	Tertiary	577	46.5 (43.8–49.3)^a^	473	38.0 (35.3–40.7)^a^
Income	<900 euros	718	66.1 (63.2–68.9)^a^	375	30.4 (27.8–33.0)^a^
	900–1,299 euros	255	23.5 (20.9–26.0)^a^	337	27.3 (24.8–29.8)^a^
	1,300–1700 euros	75	6.9 (5.4–8.4)^a^	239	19.4 (17.2–21.6)^a^
	≥1700 euros	39	3.6 (2.5–4.7)^a^	283	22.9 (20.6–25.3)^a^
Household’s financial wellbeing	Living comfortably	132	12.1 (10.2–14.1)^a^	249	20.0 (17.9–22.3)^a^
	Coping	579	52.9 (50.0–55.9)^a^	680	54.7 (51.9–57.4)^a^
	Difficult	280	25.6 (23.1–28.2)^a^	250	20.1 (17.9–22.4)^a^
	Very difficult	103	9.4 (7.8–11.3)^a^	65	5.2 (4.1–6.6)^a^
Self-rated health	Good	637	51.1 (48.3–53.9)^a^	739	59.4 (56.7–62.2)^a^
	Average	525	42.1 (39.4–44.9)^a^	409	32.9 (30.3–35.5)^a^
	Poor	84	6.7 (5.3–8.1)	95	7.6 (6.2–9.1)
Stress	No	970	78.3 (75.9–80.5)^a^	888	71.5 (68.9–74.0)^a^
	Yes	269	21.7 (19.5–24.1)^a^	354	28.5 (26.0–31.1)^a^
Depressiveness	No	899	72.3 (69.8–74.8)	933	75.3 (72.8–77.6)
	Yes	344	27.7 (25.2–30.2)	306	24.7 (22.4–27.2)
Overtiredness	No	604	48.5 (45.7–51.3)	588	47.3 (44.5–50.0)
	Yes	641	51.5 (48.7–54.3)	656	52.7 (50.0–55.5)

^a^ Statistically significant (p < 0.05) differences between column proportions.

**FIGURE 1:**
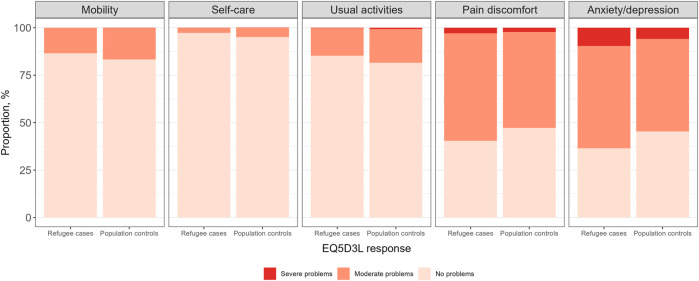
Distribution of responses by health-related quality of life dimension of the refugees versus general population (Estonia, 2024).

Figure 1 illustrates the HRQoL by EQ-5D-3L descriptive system in both study groups. Any problems with mobility were reported by 13.4% of refugees and 16.7% of controls (p < 0.05). Similarly, refugees had significantly lower proportion reporting any problems with self-care (2.9% vs. 4.9%), usual activities (14.7% vs. 18.5%), whereas a significantly higher proportion of refugees reported any problems on the pain/discomfort (59.6% vs. 52.8%; p < 0.05) and anxiety/depression (63.5% vs. 54.7%, p < 0.05) domains compared the general population. The mean EQ-5D index value in the refugee group was 0.742 (95% CI 0.732–0.752) and 0.763 (95% CI 0.752–0.774) in the general population group, i.e., the refugees had significantly (p = 0.005) lower HRQoL compared to the general population. The EQ-5D-3L index value 1 referring to perfect health state was reported by 22.6% in the refugee and 26.0% in the control group (non-significant difference); the overall distribution of EQ-5D-3L index values in both study groups is given in Supplementary Material.

In univariate regression models (Table 2), all variables considered in the study demonstrated significant association with HRQoL. Notably, refugees had significantly lower EQ-5D-3L index score (estimate −0.028, p = 0.008) compared to control group. This difference was slightly attenuated (estimate −0.028, p = 0.018) in model 2 adjusted to sex, age, marital status and education. While females and older respondents had lower HRQoL estimate, being married or cohabiting was associated with higher EQ-5D index value compared to being single. The effects of education on HRQoL were statistically non-significant in Model 2. After inclusion of income and households’ financial situation (Model 3), the difference between refugees and population controls became non-significant. While only group difference between 1,300 and 1700 vs. <900 euros was significant for income, indicator of household’s financial wellbeing demonstrated a graded association with HRQoL. This effect was heavily attenuated in Model 4 which introduced self-rated health and three mental health indicators. While these indicators were statistically significant predictors of HRQoL, variables of study group, sex, marital status, education and income were rendered statistically non-significant. After sequentially omitting marital status, education and income from the model, the difference in HRQoL by study group became statistically significant. Although the effect was relatively modest compared to health indicators included in the model, refugees had lower EQ-5D-3L index score (estimate −0.017, p = 0.029) compared to control group. In both study groups, females, older respondents, those having financial problems in the household and poorer health had lower HRQoL based on our data.

**TABLE 2:** Tobit regression models describing the association between predictor variables and health-related quality of life index score (Estonia, 2024).

Predictor variable	Model 1 (univariate)		Model 2		Model 3		Model 4		Model 5 (final)	
	Estimate	P-value	Estimate	P-value	Estimate	P-value	Estimate	P-value	Estimate	P-value
Refugees vs. controls	**−0.028**	0.008	**−0.024**	0.018	−0.001	0.932	−0.009	0.279	**−0.017**	0.029
Sex: female vs. men	**−0.049**	<0.001	**−0.039**	<0.001	**−0.030**	0.009	−0.013	0.137	**−0.017**	0.049
Age (cont.)	**−0.003**	<0.001	**−0.003**	<0.001	**−0.002**	<0.001	**−0.002**	<0.001	**−0.002**	<0.001
Marital: married/cohabiting vs. single	0.009	0.489	**0.034**	0.010	**0.029**	0.021	0.014	0.150	-	-
Marital: divorced/separated/widowed vs. single	**−0.078**	<0.001	−0.028	0.154	−0.017	0.378	−0.015	0.285	-	-
Education: secondary/vocational vs. primary	**−0.058**	0.050	−0.045	0.124	−0.054	0.056	−0.034	0.114	-	-
Education: tertiary vs. primary	−0.044	0.136	−0.026	0.386	−0.051	0.076	−0.034	0.125	-	-
Income: 900–1,299 vs. <900	**0.048**	<0.001	-	-	0.016	0.192	0.013	0.159	-	-
Income: 1,300–1700 vs. <900	**0.080**	<0.001	-	-	**0.032**	0.047	0.023	0.058	-	-
Income: >1700 vs. <900	**0.096**	<0.001	-	-	0.021	0.224	0.020	0.138	-	-
Financial wellbeing: coping vs. well off	**−0.066**	<0.001	-	-	**−0.050**	0.001	−0.005	0.631	−0.010	0.337
Financial wellbeing: finding it difficult vs. well off	**−0.160**	<0.001	-	-	**−0.136**	<0.001	**−0.036**	0.006	**−0.046**	<0.001
Financial wellbeing: Very difficult vs. well off	**−0.240**	<0.001	-	-	**−0.202**	<0.001	**−0.041**	0.025	**−0.053**	0.002
Self-rated health: average vs. good	**−0.205**	<0.001	-	-	-	-	**−0.129**	<0.001	**−0.129**	<0.001
Self-rated health: poor vs. good	**−0.402**	<0.001	-	-	-	-	**−0.252**	<0.001	**−0.254**	<0.001
Stress: yes vs. no	**−0.210**	<0.001	-	-	-	-	**−0.067**	<0.001	**−0.068**	<0.001
Depressiveness: yes vs. no	**−0.217**	<0.001	-	-	-	-	**−0.077**	<0.001	**−0.077**	<0.001
Overtiredness: yes vs. no	**−0.219**	<0.001	-	-	-	-	**−0.098**	<0.001	**−0.098**	<0.001
*(Intercept)*	*na*	*na*	***0.938***	*<0.001*	***0.975***	*<0.001*	***1.046***	*<0.001*	***1.044***	*<0.001*
*Log(scale)*	*na*	*na*	*−1.468*	*<0.001*	*−1.508*	*<0.001*	*−1.790*	*<0.001*	*−1.786*	*<0.001*
*Log-likelihood*	*na*	*na*	*−466.8*	​	*−393.4*	​	*137.7*	​	*130.4*	​
*d.f*	*na*	*na*	*9*	​	*15*	​	*20*	​	*13*	​

Bold values represents the P-value < 0.05. Italic values represents Additional model parameters.

## Discussion

Using sex- and age-matched survey data on HRQoL of Ukrainian refugees and of Estonian general population, the results demonstrated significant HRQoL differences between the study groups. While refugees reported less problems with mobility, self-care and usual activities, a higher prevalence of pain/discomfort and anxiety/depression was found compared to the control group. The differences in reported problems on the HRQoL dimensions translated into slightly lower EQ-5D-3L index score among Ukrainian refugees. This difference in EQ-5D-3L index score persisted after adjusting for a range of socio-demographic and health-related indicators that demonstrated significant variation between the study groups.

Before discussing these findings in detail, some aspects regarding the data and methods should be considered. While the declining response rates are universal challenge in survey research and also documented for HBEP study [19], resulting non-response bias might have affected the data. This is evident in the male-to-female ratio in the refugee survey, which declined from 0.48 in the sample to 0.31 in the data and contrasts with corresponding 0.72 ratio in HBEP data. However, predominant share of women among adult refugees is a common indication of the migrant population fleeing a conflict and conveys, in addition to demographic variations, also distinct epidemiological characteristics. The use of sex- and age-matching was thus an important measure to mitigate the effects of demographic variation in two datasets. However, as the refugee-matched control group has a different demographic structure, the current results cannot be directly generalized to Estonian general population. Secondly, while SHURE study included wide range of indicators addressing the migratory background, change in living conditions etc., corresponding indicators were not present in the HBEP questionnaire and direct comparison was limited only variables available in both datasets. However, despite the inclusion of key socio-demographic and health indicators, it is unlikely that the set of variables accounts for total variance in the dependent variable. Thus, potential residual bias should be considered when interpreting the results. Third, the modelling strategy might have also affected the results. We have conducted a sensitivity analysis using OLS regression which yielded very similar results across all models (see Supplementary Material). Also, models used stepwise removal of non-significant variables which might yield biased coefficients and p-values [24]. However, the choice of variables to the analysis reflected those that all data is based on self-reports and could not be validated externally. This might have impact on the results either due to underreport or overreport certain health conditions, particularly mental health symptoms as found in previous study [25]. All these considerations are inherent to studies employing similar designs and, as such, do not represent unique or additional limitations to the present study.

The key finding is the persistence of lower HRQoL among refugee group, even after adjusting for a range of socio-demographic and health-related covariates. However, the difference (−0.017 EQ-5D units) compared to the population controls was very minor. Based on the findings of a recent systematic review [26], the minimally important difference was −0.02 for deteriorated EQ-5D-3L scores. While the lower EQ-5D index score might not convey clinically significant difference in HRQoL, these results still suggests that refugee status itself may entail unique and enduring stressors that are not adequately captured by demographic or health indicators. Despite similar or slightly better outcomes in physical health domains such as mobility, self-care, and usual activities, refugees exhibited significantly higher levels of problems in the EQ-5D dimensions related to pain/discomfort and anxiety/depression. This corresponds to an earlier systematic review on refugee HRQoL using WHOQOL-Bref instrument [6], where refugees had higher scores for the physical and lower scores for psychological domain compared to general population. In our data, the observed difference in the anxiety/depression domain (63.5% vs. 54.7%) may reflect both pre-migration stressors such as exposure to armed conflict and loss, as well as post-migration challenges including acculturation stress, housing insecurity, and separation from family members [27, 28]. The latter might be reflected in the distribution of marital status variable, where the refugee population had significantly higher share of divorced/widowed and lower proportion of married/cohabiting individuals compared to population controls. Similarly, a recent study on Ukrainian refugees in Poland [29] reported acute stress prevalence exceeding 90% which is indicative of high level of trauma. Although data on post-traumatic stress disorder was not available for general population, 79.6% of refugees in SHURE data had experienced traumatic experience [30]. This contrasts with the lower reporting of perceived stress among refugees compared to population controls (21.7% vs. 28.5%, p < 0.05). It is also plausible that the difference in perceived stress prevalence might stem from peer comparison used in the response options where categories “yes, more than people on the average” or “yes, but no more than people on the average” might have different baseline for refugees and population controls. Also, it is likely that refugees from active conflict zones may be more likely to somatize psychological distress or may have varying thresholds for labelling emotional discomfort as a mental health concern. While the differential item reporting has also potential implications for EQ-5D-3L assessments, the specific topic warrants further research but is out of the scope of the current study.

The results also emphasize that refugees do not exhibit uniformly worse HRQoL compared to host population. In our data, higher proportion of refugees reported no problems on EQ-5D-3L dimensions mobility, self-care, and usual activities. One potential explanation for this finding is the “healthy migrant effect” that suggest individuals who are healthier or more resilient are more likely to migrate [31]. While it is mostly applicable to voluntary migration, a positive self-selection may also be present among refugees, particularly those who undertake arduous journeys or who are resettled through official humanitarian programs requiring minimal health screening [32]. In the context of Ukrainian refugee crisis, a recent study from Italy [33] also reported lower non-communicable disease prevalence among registered refugees compared to rates usually found in the Ukraine population. Indirect support for this argument in our data relates to educational variation where refugees had substantially higher proportion of tertiary education compared to controls (46.5% vs. 38.0%) suggesting that younger, more mobile, but also physically healthier individuals were more likely to emigrate. This is also supported by another recent study in Estonia where 60% of refugees were in ages 18–59 years and individuals >60 years accounted for <10% of refugees [18]. However, this advantage in physical health may be short-lived, as psychosocial stressors due to displacement, unemployment and other similar factors often contribute to health deterioration over time [34]. Consistent with broader public health literature, this study found that both economic vulnerability and perceived poor health were strongly associated with reduced EQ-5D-3L index scores in both study groups. Although the effects of education and income became non-significant after inclusion of health variables in our regression model, the relative measure on household income remained a strong predictor of HRQoL. Based on SHURE survey data [30], 34.6% of refugees in Estonia reported financial problems, substantial increase from 15.1% before emigration. The key priority needs for refugee households from Ukraine in Estonia are the need to secure employment/livelihoods support (32%), language courses (33%), access to healthcare services (22%) and training of adults (17%) [18]. Therefore, in order to maintain and improve health of the refugees, concentrated intersectoral efforts are needed that address the wider social determinants of health, such as income security, housing, employment, and access to healthcare.

These findings contribute to the literature on refugee HRQoL and extend the knowledge on HRQoL of Ukrainian refugees in the context of ongoing refugee crisis in Europe. The findings underline the complex nature of refugee health and offer a nuanced view of both the vulnerabilities and potential resilience factors in this population. The findings highlight the importance of addressing mental health needs and socioeconomic stressors through integrated interventions. Future longitudinal research is needed to monitor changes in HRQoL over time and evaluate the effectiveness of policy interventions in mitigating health disparities between refugee and host populations.
