# Supplementary material for: Health-Related Quality of Life Among Ukrainian War Refugees Compared to the General Population in Estonia
Source: Int J Public Health. 2026 Feb 5;71:1608807. doi: 10.3389/ijph.2026.1608807 (PMC12916432; doi:10.3389/ijph.2026.1608807)
Supplement: Supplementary file 1 [file Supplementaryfile1.docx]

**Supplementary materials**

Figure S1. Distribution of EQ-5D-3L index scores in both study groups


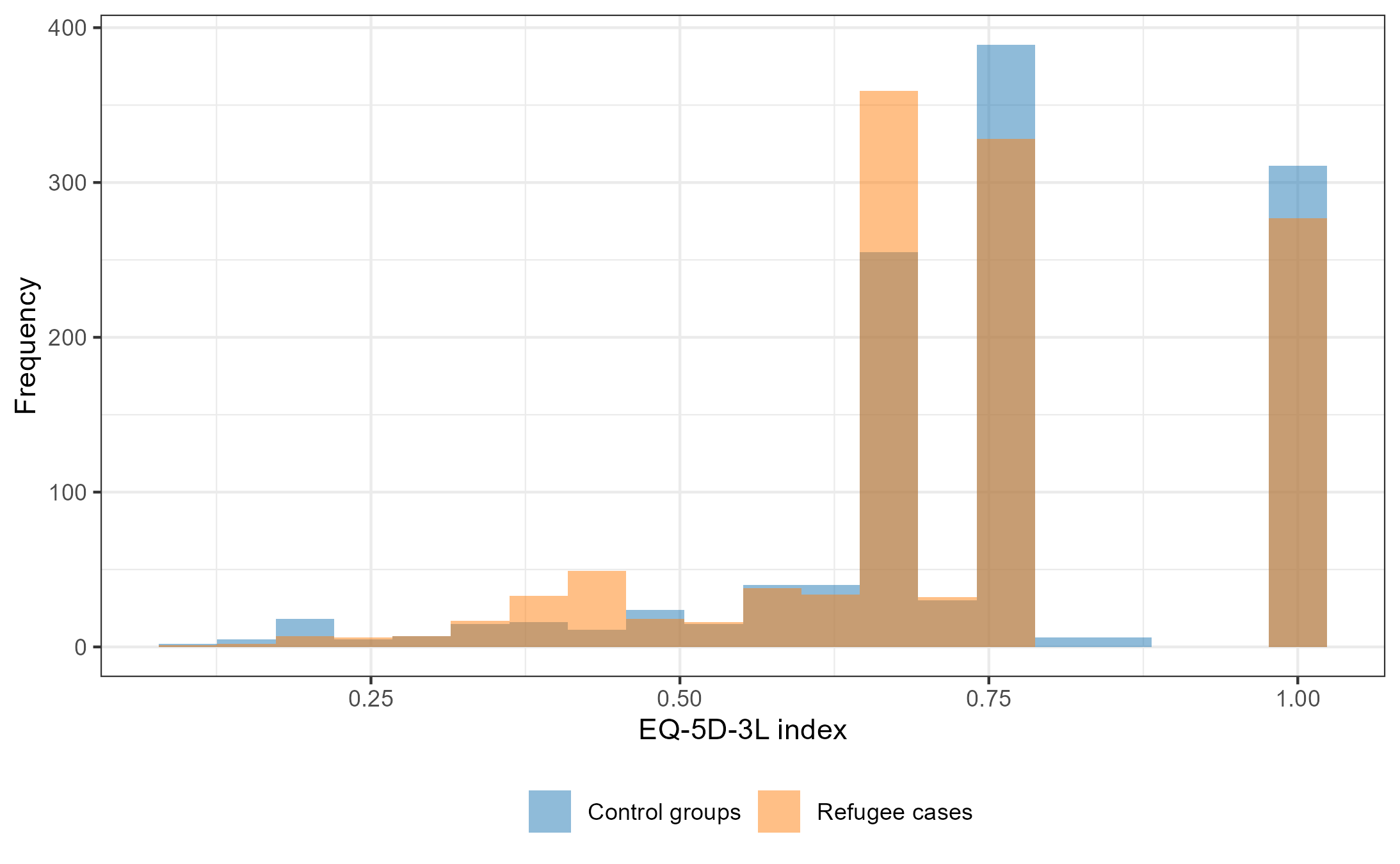


**Table S2.** Comparison of Tobit and OLS regression models (p < 0.05 in **bold**) describing the association between predictor variables and EQ-5D index score

|  | **Model 1 (univariate) estimates** | | **Model 2 estimates** | | **Model 3 estimates** | | **Model 4 estimates** | | **Model 5 (final) estimates** | |
| --- | --- | --- | --- | --- | --- | --- | --- | --- | --- | --- |
|  | **Tobit** | **OLS** | **Tobit** | **OLS** | **Tobit** | **OLS** | **Tobit** | **OLS** | **Tobit** | **OLS** |
| **Refugees vs controls** | **-0.028** | **-0.022** | **-0.024** | **-0.020** | -0.001 | -0.001 | -0.009 | -0.009 | **-0.017** | **-0.015** |
| Sex: female vs men | **-0.049** | **-0.035** | **-0.039** | **-0.275** | **-0.030** | **-0.020** | -0.013 | -0.010 | **-0.017** | -0.012 |
| Age (cont.) | **-0.003** | **-0.002** | **-0.003** | **-0.002** | **-0.002** | **-0.002** | **-0.002** | **-0.001** | **-0.002** | **-0.001** |
| Marital: married/cohabiting vs single | 0.009 | 0.010 | **0.034** | **0.030** | **0.029** | **0.026** | 0.014 | 0.013 | - | - |
| Marital: divorced/separated/widowed vs single | **-0.078** | **-0.060** | -0.028 | -0.021 | -0.017 | -0.011 | -0.015 | -0.011 | - | - |
| Education: secondary/vocational vs primary | **-0.058** | -0.039 | -0.045 | -0.029 | -0.054 | -0.036 | -0.034 | -0.022 | - | - |
| Education: tertiary vs primary | -0.044 | -0.026 | -0.026 | -0.012 | -0.051 | -0.032 | -0.034 | -0.020 | - | - |
| Income: 900-1299 vs <900 | **0.048** | **0.040** | - | - | 0.016 | 0.014 | 0.013 | 0.011 | - | - |
| Income: 1300-1700 vs <900 | **0.080** | **0.063** | - | - | **0.032** | **0.025** | 0.023 | 0.017 | - | - |
| Income: >1700 vs <900 | **0.096** | **0.077** | - | - | 0.021 | 0.019 | 0.020 | 0.016 | - | - |
| Comfort: coping vs well off | **-0.066** | **-0.049** | - | - | **-0.050** | **-0.036** | -0.005 | -0.003 | -0.010 | -0.008 |
| Comfort: finding it difficult vs well off | **-0.160** | **-0.125** | - | - | **-0.136** | **-0.107** | **-0.036** | **-0.030** | **-0.046** | **-0.037** |
| Comfort: very difficult vs well off | **-0.240** | **-0.202** | - | - | **-0.202** | **-0.171** | **-0.041** | **-0.041** | **-0.053** | **-0.052** |
| Self-rated health: average vs good | **-0.205** | **-0.163** | - | - | - | - | **-0.129** | **-0.104** | **-0.129** | **-0.104** |
| Self-rated health: poor vs good | **-0.402** | **-0.357** | - | - | - | - | **-0.252** | **-0.234** | **-0.254** | **-0.237** |
| Stress: yes vs no | **-0.210** | **-0.177** | - | - | - | - | **-0.067** | **-0.062** | **-0.068** | **-0.063** |
| Depressiveness: yes vs no | **-0.217** | **-0.179** | - | - | - | - | **-0.077** | **-0.063** | **-0.077** | **-0.064** |
| Overtiredness: yes vs no | **-0.219** | **-0.169** | - | - | - | - | **-0.098** | **-0.071** | **-0.098** | **-0.071** |
| *(Intercept)* | *na* | *na* | ***0.938*** | ***0.865*** | ***0.975*** | ***0.891*** | ***1.046*** | ***0.962*** | ***1.044*** | ***0.967*** |
